# Supplementary material for: Mechanisms governing the pioneering and redistribution capabilities of the non-classical pioneer PU.1
Source: Nat Commun. 2020 Jan 21;11:402. doi: 10.1038/s41467-019-13960-2 (PMC6972792; doi:10.1038/s41467-019-13960-2)
Supplement: Supplementary file 7 — Source data [file 41467_2019_13960_MOESM7_ESM.zip › Source_Data/FigureS2/FigureS2B_MotifScanOutput/KmeansCluster6/homerResults/motif2.similar.html]

motif2

## Information for motif2

C
G
T
A
A
T
C
G
A
C
G
T
A
C
G
T
A
G
T
C
A
G
C
T
A
G
T
C
  
Reverse Opposite:  

A
C
T
G
C
T
G
A
A
C
T
G
C
G
T
A
C
G
T
A
A
T
G
C
A
C
G
T
  

|  |  |
| --- | --- |
| p-value: | 1e-346 |
| log p-value: | -7.989e+02 |
| Information Content per bp: | 1.963 |
| Number of Target Sequences with motif | 1559.0 |
| Percentage of Target Sequences with motif | 47.34% |
| Number of Background Sequences with motif | 6907.4 |
| Percentage of Background Sequences with motif | 16.06% |
| Average Position of motif in Targets | 102.3 +/- 54.1bp |
| Average Position of motif in Background | 101.1 +/- 76.9bp |
| Strand Bias (log2 ratio + to - strand density) | 0.0 |
| Multiplicity (# of sites on avg that occur together) | 1.09 |
| Motif File: | file (matrix) reverse opposite |

### Similar de novo motifs found

|  |  |  |  |  |  |  |  |
| --- | --- | --- | --- | --- | --- | --- | --- |
| Rank | Match Score | Redundant Motif | P-value | log P-value | % of Targets | % of Background | Motif file |
| 1 | 0.835 | A C T G C T G A A T G C C A G T G C A T G T A C A G C T A T G C C G A T G C A T | 1e-192 | -443.684936 | 28.73% | 9.47% | motif file (matrix) |
| 2 | 0.762 | G C T A A C T G G C A T C A G T T A G C G A C T G A T C C T G A G A C T C T A G C G T A | 1e-70 | -161.469003 | 6.56% | 1.30% | motif file (matrix) |
